# Is the prevalence of pre-eclampsia affected by HIV/AIDS? A retrospective case–control study

**DOI:** 10.5830/CVJA-2012-078

**Published:** 2013-03

**Authors:** VMS Kalumba, J Moodley, TD Naidoo

**Affiliations:** Department of Obstetrics and Gynaecology, Pietermaritzburg Complex of Academic Hospitals; Women’s Health and HIV Research Group, Department of Obstetrics and Gynaecology, University of KwaZulu-Natal, Durban, South Africa; Department of Obstetrics and Gynaecology, Pietermaritzburg Complex of Academic Hospitals; Women’s Health and HIV Research Group, Department of Obstetrics and Gynaecology, University of KwaZulu-Natal, Durban, South Africa; Department of Obstetrics and Gynaecology, Pietermaritzburg Complex of Academic Hospitals; Women’s Health and HIV Research Group, Department of Obstetrics and Gynaecology, University of KwaZulu-Natal, Durban, South Africa

**Keywords:** HIV, CD_4_ count, pre-eclampsia, eclampsia, pregnancy

## Abstract

**Objective:**

To evaluate the rate of HIV/AIDS (and CD_4_ levels) in women with pre-eclampsia compared to that of a control group.

**Methods:**

This was a retrospective case–control study in a tertiary and regional hospital in South Africa. We reviewed the hospital records of women who had delivered in these hospitals between 1 January 2008 and 30 June 2010. The records of women with pre-eclampsia during the study period were analysed. Their HIV infection rate was compared to that of a control group consisting of normotensive healthy pregnant women.

**Results:**

Among 492 cases of pre-eclampsia, 130 (26.4%) were HIV infected. In the control group, 183/500 (36.6%) were HIV infected (*p* = 0.001, OR = 0.62, 95% CI: 0.47–0.82). After adjustment to match the difference in maternal age and parity, the rate of HIV/AIDS was lower in the pre-eclamptic group than in the control group (*p* = 0.005, OR = 0.658).

**Conclusion:**

The rate of HIV/AIDS was significantly lower in women with pre-eclampsia than in normotensive healthy pregnant women.

## Abstract

Pre-eclampsia, a condition unique to human pregnancy, clinically presents with hypertension and proteinuria after the 20th week of gestation. It complicates 7–10% of pregnancies worldwide and is a major cause of maternal and perinatal morbidity and mortality.^1^,^2^ The current understanding of the aetiology of pre-eclampsia remains unclear.^1^ It has been proposed that placental maladaptation leads to decreased utero-placental blood flow and subsequent intracellular hypoxia, resulting in the release of various substances including trophoblastic debris and apoptotic cells. These cause an imbalance between anti-angiogenic and angiogenic factors, resulting in widespread multi-organ endothelial dysfunction.^2^ The end result is generalised vasospasm, hypertension and multiple organ affectation.^3^

Although much of the pathophysiology of pre-eclampsia is known, the current debate is what causes the placental maladaptation. It is believed that immunological factors may be involved in initiating the cascade of events mentioned above.^2^,^4^ Also, pre-eclampsia has been shown to represent an excessive generalised maternal sterile inflammatory response to pregnancy.^5^,^6^ Further, it has been postulated that the frequency of pre-eclampsia may be affected by immunosuppressive conditions such as HIV/AIDS.^4^,^7^-^9^

Data on the impact of HIV on the rate of pre-eclampsia are conflicting. There is no consensus as to whether HIV-infected women are at a lower, equal or higher risk of developing pre-eclampsia than the general population. Most studies have included small sample sizes and/or have been retrospective chart reviews.^8^

In South Africa, approximately 30% of antenatal patients are infected with HIV.^10^ Also, non-pregnancy related infections (mainly HIV/AIDS) and hypertensive disorders are the commonest causes of maternal mortality and morbidity.^11^ Hence, South Africa represents an ideal site for a study involving HIV and pre-eclampsia.

The aim of this study was to evaluate the association between HIV infection and pre-eclampsia. To test the hypothesis that women with pre-eclampsia are less likely to be affected by HIV/AIDS, the rate of HIV in pre-eclamptics was compared to that of a control group without pre-eclampsia. In addition, the CD_4_ count levels between the two groups were compared to test the hypothesis that immune-suppression could have a protective effect against pre-eclampsia.

## Methods

This was a retrospective case–control study conducted at Grey’s and Edendale hospitals (a tertiary and a regional hospital, respectively) in Pietermaritzburg, South Africa. The maternity birth registries in the two study sites were reviewed.

Women who had delivered between January 2008 and June 2010 with a diagnosis of pre-eclampsia were identified and their hospital files were retrieved from the medical registry. Those meeting the inclusion criteria were selected and all relevant data were collected on a structured data sheet until the estimated sample size was reached. The inclusion criteria were a diagnosis of pre-eclampsia, known HIV status, singleton pregnancy and no evidence of other chronic medical conditions, namely hypertension, diabetes, renal disease and connective tissue disease.

The exclusion criteria were women with unknown/unrecorded HIV status, multiple pregnancy and chronic hypertension. Subsequently, an equivalent number of women without pre-eclampsia (control group) were randomly selected to match the case criteria. With the exception of the diagnosis of pre-eclampsia, the inclusion and exclusion criteria of the controls were similar to those of the cases.

The SOMANZ (Society of Obstetric Medicine of Australia and New Zealand) classification and definitions of hypertensive disorders of pregnancy were used.^12^ However, for this research purpose, the diagnosis of pre-eclampsia was based only on hypertension and proteinuria from 20 weeks of gestation. Proteinuria was considered as urine dipstick protein of 1+ or more (on two occasions at least) or a 24-hour urine protein of at least 300 mg. In addition, only women whose high blood pressure had returned to normal values within a week of delivery were included in the study to rule out chronic hypertension.

All women are offered counselling and voluntary testing for HIV at these hospital sites as the standard of care. Institutional ethical and hospital regulatory permission was obtained for the study (Biomedical Research Ethics Committee, University of KwaZulu-Natal, South Africa; reference number BE 151/010).

In the province of KwaZulu-Natal, the HIV/AIDS infection rate in pregnant women is 40%.^10^ Assuming a reduction in HIV rate from 40% in controls to 25% in cases (pre-eclamptics), 890 women (445 cases and 445 controls) were required to achieve a study power of 80% with statistical significance of *p* < 0.05. This sample size was also estimated by assuming that the proportion of HIV-infected women with a CD_4_ cell count < 200 cells/μl (immune-compromised) would be lower among pre-eclamptics.

## Statistical analysis

SPSS version 18 was used to analyse the data. A *p*-value < 0.05 was considered statistically significant. Pearson’s Chi-square tests were used to compare categorical variables between cases and controls, while *t*-tests were used to compare quantitative variables between the two groups if the data were normally distributed. Mann–Whitney tests were used if the data were skewed. Binary logistic regression analysis was conducted in order to assess the adjusted odds ratio for HIV status according to the age and parity difference between the groups.

## Results

There was a total of 23 988 deliveries over the study period at the two study sites. Among them, 1 892 women were identified with a diagnosis of pre-eclampsia (including imminent eclampsia, and eclampsia).

Data were collected from 500 cases (pre-eclamptics) and 500 controls (normotensive healthy pregnant women) who met the inclusion criteria. Among the pre-eclamptics, eight cases had information missing from their files (birth weight and/or gestational age at delivery) and were therefore excluded. Finally, 492 cases were used for analysis. The maternal age of the two groups are shown in Table 1.

**Table 1:** Maternal Age Distribution In Cases And Control Group

*Age*	*< 20 years, n (%)*	*20–29 years, n (%)*	*30–39 years, n (%)*	*≥ 40 years, n (%)*	*Mean age (years)*	*Total, n (%)*
Controls	147 (29.4)	210 (42.0)	116 (23.2)	27 (5.4)	25.25	500 (100%)
Pre-eclamptics	145 (20.4)	250 (50.6)	93 (18.8)	6 (1.2)	24.09	492 (100%)
Total	292 (29.4)	460 (46.3)	209 (21.9)	33 (3.3)	24.67	992 (100%)

The rate of HIV infection in the pre-eclamptic group was 26.4%. In the control group, the HIV infection rate was 36.6% (OR = 0.62, 95% CI: 0.47–0.82, *p* = 0.001) (Table 2).

**Table 2:** HIV Rate In Cases And Control Group

	*HIV positive n (%)*	*HIV negative n (%)*	*Total n (%)*
Control group	183 (36.6)*	317 (63.4)	500 (100)
Pre-eclamptic group	130 (26.4)*	362 (73.6)	492 (100)
Total	313 (31.6)	679 (68.4)	992 (100)

*p = 0.001; OR = 0.62; 95% CI: 0.47–0.82.

Pre-eclamptic women were 38% less likely to be HIV infected than the control group without pre-eclampsia. Because the cases and controls were not exactly age and parity matched, the difference between them in HIV infection rate was adjusted for this confounding factors using logistic regression analysis. The odds ratio of being a case (pre-eclamptic) compared to a control was 0.658 for HIV negative (*p* = 0.005) after adjustment. This means that HIV-infected women were 34.2% less likely to develop pre-eclampsia than women not infected with HIV.

The results of the CD_4_ counts were available in only 66 cases (pre-eclamptics) and 75 controls.

In women with pre-eclampsia, the median CD_4_ count was 304 cells/μl with a maximum of 906 cells/μl and a minimum of 10 cells/μl, versus 208 cells/μl with a maximum of 725 cells/μl and a minimum of 56 cells/μl in the control group (*p* = 0.008). The proportion of pre-eclamptic women with ≥ 3+ protein was higher in the HIV-negative group (39.2%) than in the HIV-positive group (27.9%) (*p* = 0.022).

## Discussion

As far as we know, this is the first study to report the rate of HIV infection in women with pre-eclampsia in comparison with a control group without pre-eclampsia. Most studies on HIV and pre-eclampsia have compared the rate of pre-eclampsia between uninfected and HIV-infected women.^4^,^7^-^9^

The rate of HIV/AIDS infection was lower in pre-eclamptic women than in the control group. These findings suggest that women with pre-eclampsia are less likely to be affected by HIV infection than the general population. In other words, HIV infection being the exposure and pre-eclampsia being the outcome variable, HIV-infected women are at a lower risk of developing pre-eclampsia. Our findings also suggest that HIV infection could have a protective effect against the development of pre-eclampsia.

The underlying mechanism of the protective effect of HIV infection is unclear. As postulated in our hypothesis, it is possibly associated with immune suppression in HIV-infected women. To further evaluate this association, the level of immunity (as expressed by the CD_4_ count) between the two groups was compared. The CD_4_ count result, however, was available in only 66 cases and 75 controls. The median CD_4_ count was lower in the control group without pre-eclampsia (median CD_4_ count = 208 cells/μl) than in the pre-eclamptic women (median CD_4_ count = 304 cells/μl) (*p* = 0.008). This suggests that among HIV-infected women, the immunity was less affected in those who developed pre-eclampsia.

We also found that the proportion of pre-eclamptic women with +3 protein or more in their urine dipstick was higher in the HIV-negative group (39.2%) than in the HIV-positive group (27.9%) (*p* = 0.022). This correlates with the fact that the mean serum total protein and albumin levels were lower in the HIV-negative than the HIV-positive group (*p* < 0.0001, *p* = 0.013, respectively) and could suggest that immunity plays a role in the pathogenesis of proteinuria in pre-eclampsia.

Our findings are different from those of Frank *et al*. who found no significant association between HIV infection and pre-eclampsia; the rate of pre-eclampsia in their study was not different between HIV-infected (5.7%) and uninfected women (5.2%). In their study the CD_4_ count was known in only 13 cases out of 704 HIV-infected women.^7^

The main difference between our study and that of Frank *et al.* is the fact that they included women with underlying medical conditions.^7^ This group with underlying medical conditions may have had other independent risk factors for pre-eclampsia and the immune maladaptation was less likely to be the initial event. Underlying medical conditions constituted an exclusion criterion from our study.

Also, the power was estimated assuming a reduction in the rate of pre-eclampsia from 8% (in uninfected women) to 5% in HIV-infected women. This margin was very narrow if we consider the wide variability in the rate of pre-eclampsia from one geographical area to another, and even from one period of time to another in the same area. The findings can easily swing for or against the hypothesis. Our findings are less likely to have been affected by minimal variations in the rate.

Frank *et al*. did raise the fact that the pre-term birth rate is high in HIV-infected women and it is possible that a proportion may have delivered prior to the onset of pre-eclampsia.^7^ Data on the rate of pre-term births in HIV-infected women is, however, conflicting with some studies showing no differences between HIV-infected and uninfected women.^12^,^13^ This confounding factor is less likely to have affected our results since the mean gestational age at delivery was not significantly different between uninfected and HIV-infected women (34.86 weeks and 33.65 weeks, respectively).

The AmRo study found no difference in pre-eclampsia rate between HIV-positive and HIV-negative women.^12^ The incidence of pre-eclampsia was 2.8% among 143 HIV-positive women. This sample size was too small to demonstrate a possible statistical difference and 93 out of 143 women were already on HAART (highly active antiretroviral therapy), hence they were possibly immune competent.^12^

Suy *et al*.^13^ found a very low rate of pre-eclampsia among 258 HIV-infected women who were not on HAART. In 140 women on HAART, however, the pre-eclampsia rate was significantly higher (11%). In the same group, the rate of pre-eclampsia in uninfected women was 2.8%. These results suggested that HIV-infected women are at a lower risk of developing pre-eclampsia than uninfected women, but at a higher risk when on HAART.^13^

In our study, we could not evaluate for replication of the rate of pre-eclampsia in women on HAART because of its different approach. However, the findings also suggested that immunosuppression could be protective against pre-eclampsia. Immune reconstitution could alleviate this protection and even possibly increase the risk of developing pre-eclampsia.

Wimalasundera *et al*.^9^ also found a low rate of pre-eclampsia in HAART-naïve, HIV-infected women but a higher rate in those on HAART. A retrospective study by Mattar *et al*.^4^ found a low rate of pre-eclampsia among 123 HIV-positive women (0.8%) compared to 1 708 controls (10.6%); this was a significant difference. The median CD_4_ count in HIV-infected women was 531 cells/μl.

As illustrated above, the results from various studies are conflicting. This is probably due to differences in study design and approach. Some studies included patients with underlying chronic medical conditions.^4^,^9^

Our approach was unique in comparing the rate of HIV infection in pre-eclamptic women with that in a control group. Because of the high rate of HIV in South Africa, this is the most important study so far on HIV and pre-eclampsia. We excluded women with underlying chronic medical conditions and evaluated the level of immunity as per the CD_4_ count level, and correlated proteinuria and other parameters of pre-eclampsia with the HIV status.

There were some limitations to our study and this included the fact that it was a retrospective study. The CD_4_ counts were available in only a few cases of both pre-eclamptics and the control group (small sample size). Also, in many cases, these were not recent results; the testing had been carried out up to six months earlier. In these cases, this did not reflect the actual immune status of the women at the time of recruitment.

For the same reason, we could not make a correlation between the severity of proteinuria and the level of immunity as expressed by the CD_4_ count. A more accurate quantification of proteinuria (by 24-hour urine protein levels or spot protein:creatinine ratio) and recently obtained CD_4_ counts could provide a better evaluation.

## Conclusion

Our study revealed a significantly lower rate of HIV/AIDS infection in pre-eclamptic women compared to those without pre-eclampsia. This finding suggests that women with HIV/AIDS are less likely to develop pre-eclampsia.
